# Novel Al-Ce-C-O-Mg Grain Refiners with Superior Efficiency and Mechanical Properties Enhancement for AZ91 Alloys

**DOI:** 10.3390/ma18204782

**Published:** 2025-10-20

**Authors:** Juan Li, Xinfang Zhang, Wenxue Fan

**Affiliations:** 1 Zhengzhou Key Laboratory of Environmental Functional Materials, Zhengzhou University of Aeronautics, Zhengzhou 450046, China; cflijuan@zua.edu.cn; 2 Henan Key Laboratory of Aeronautical Materials and Application Technology, Zhengzhou University of Aeronautics, Zhengzhou 450046, China; teaoren@163.com; 3 School of Mechanical and Automotive Engineering, Ningbo Institute of Technology, Ningbo 315336, China

**Keywords:** AZ91 Mg alloy, grain refinement, microstructure evolution, mechanical properties

## Abstract

Grain refinement represents a critical approach in optimizing the as-cast microstructure of magnesium alloys, playing a pivotal role in the development of high-performance magnesium alloys. In the present research, a novel Al-Ce-C-O-Mg grain refiner was fabricated using an innovative rolling-assisted process, and the influence of the grain refiner on the grain size evolution of as-cast AZ91 alloy was systematically examined. The Al-Ce-C-O-Mg grain refiner prepared by the rolling-assisted process contains two types of effective refining particles—MgAl_2_O_4_ and Al_4_C_3_. These particles can act as potent nucleation sites for α-Mg in the melt, promoting efficient nucleation and achieving significant grain refinement. By adding 1.0 wt.% of the Al-Ce-C-O-Mg grain refiner to the AZ91 alloy, the grain size of the original AZ91 alloy was reduced by 73%. Moreover, adding a refiner facilitated the transformation of the coarse β-Mg_17_Al_12_ phase morphologies into a more uniformly distributed and dispersed form. The addition of 1.0 wt.% Al-Ce-C-O-Mg grain refiner to the AZ91 alloy resulted in significant improvements in its mechanical properties. The ultimate tensile strength (UTS), yield strength (YS), and elongation (EL) increased from 158 MPa, 104 MPa, and 3.9% to 203 MPa, 121 MPa, and 6.3%, respectively. The grain refiner developed in this study demonstrates promising potential for application in Mg alloys.

## 1. Introduction

Magnesium alloys are increasingly recognized as promising materials for a wide range of applications, particularly in the automotive, electronics, and aerospace industries, due to their lightweight nature, high specific strength and stiffness, and excellent castability [1,2]. However, as the demand for magnesium alloys increases, there is a growing need for alloys with enhanced performance. A key factor influencing the mechanical properties and processing characteristics of magnesium alloys is their grain size, which has been extensively documented in the literature [3]. Grain refinement is therefore regarded as a principal metallurgical approach for improving the quality of cast components. This strategy effectively suppresses the formation of coarse and columnar grain morphologies, mitigates solidification-related defects such as hot tearing and macro-segregation, and facilitates improved performance during subsequent thermomechanical processing [4]. Among the available refinement methodologies, the addition of engineered grain refiner containing potent heterogeneous nucleation substrates—such as TiB_2_ [5], TiC [6], AlB_2_ [7], Al_4_C_3_ [8], and ZnO [9]—has become the most prevalent practice in industrial Mg alloy casting owing to its efficiency, reproducibility, and compatibility with existing foundry operations.

Al-Ti-B series master alloys are widely recognized as effective, economical, and industrially convenient grain-refining agents for magnesium alloys [10]. However, despite the widespread commercial adoption of both Ti-B and Ti-C series grain refiners, several inherent limitations constrain their broader applicability. For Al-Ti-B series refiners, the refinement efficiency can be adversely affected by the agglomeration and sedimentation of TiB_2_ particles during processing, leading to non-uniform particle distribution within the melt. Furthermore, their effectiveness in Zr-containing magnesium alloys is markedly reduced due to the so-called “poisoning effect” associated with zirconium, which interferes with heterogeneous nucleation [11]. In contrast, Al-Ti-C master alloys have been extensively investigated as alternative grain refiners due to their high efficiency and environmental compatibility [12]. Adding Al-Ti-C to Mg-Al alloys essentially functions as a carbon inoculation treatment, with the refinement mechanism explained by the dual-phase nucleation theory involving Al_4_C_3_-Al_8_(Mn, Fe)_5_-α-Mg [13]. Recent studies have verified that Al_4_C_3_ particles can directly act as potent nucleation substrates for α-Mg during solidification [14]. In previous work, the influence of carbon-containing particles on the refinement of Al-containing Mg alloys was systematically examined, providing further insights into the nucleation behavior associated with this class of refiners. The results proved that Al_4_C_3_ phases can serve as nucleus cores for α-Mg [12]. On the other hand, oxide particles have the advantages of readily available raw materials, an excellent refining effect, and resistance to refinement deterioration. It has promising application prospects in refining Al-containing Mg alloys. Over the past decade, interest has increasingly focused on the use of oxides, such as ZnO [9], CaO [15], and MgO [16], as nucleation sites in Mg alloys. Among them, the low-cost and stable MgO has attracted the focused attention of scientists. Fan et al. discovered that MgO could transform to MgAl_2_O_4_ particles as a nucleation site for α-Mg when they performed the MgO inoculation treatment on Mg-Al alloy, effectively refining the grains of Al-containing Mg alloy [17]. Liao et al. found that MgAl_2_O_4_ spinel powder can effectively refine the grain size of Al-containing Mg alloys and has excellent anti-fading refinement [18].

Based on the preceding analysis, MgAl_2_O_4_ and Al_4_C_3_ particles demonstrate superior grain refinement capabilities, indicating their strong potential for widespread application as effective refining agents in magnesium alloy processing. In addition, a rare-earth element with high chemical activity, cerium (Ce), exhibits a dual function in the grain refinement process. During the preparation of grain refiners, Ce effectively improves the interfacial wettability among constituent elements, which favors the formation of particle phases. Furthermore, within Mg alloys, Ce imposes a significant growth restriction on Mg grains, thereby contributing to enhanced grain refinement. However, it is not wise to add MgAl_2_O_4_ and Al_4_C_3_ powder directly to the Mg melt to achieve refinement. When the powder is added directly to the metal melt, agglomeration and settling of the particles occur due to poor wettability, which not only weakens the refining effect, but also damages the mechanical properties. Incorporating a grain refiner containing heterogeneous nucleation particles has emerged as a practical and industrially viable approach for promoting grain refinement in magnesium alloys.

In the present study, a novel Al-Ce-C-O-Mg master alloy was developed through a newly designed preparation route, yielding in situ-formed MgAl_2_O_4_ and Al_4_C_3_ particles within the alloy matrix. The thermodynamic mechanisms governing the formation of these phases in the Al-Ce-C-O-Mg system were systematically analyzed. Furthermore, the influence of the Al-Ce-C-O-Mg master alloy on the microstructural evolution of AZ91 alloy during solidification was examined, with particular attention paid to its refinement efficiency and the consequent enhancement in mechanical performance.

## 2. Experimental Procedure

**Figure 1:**
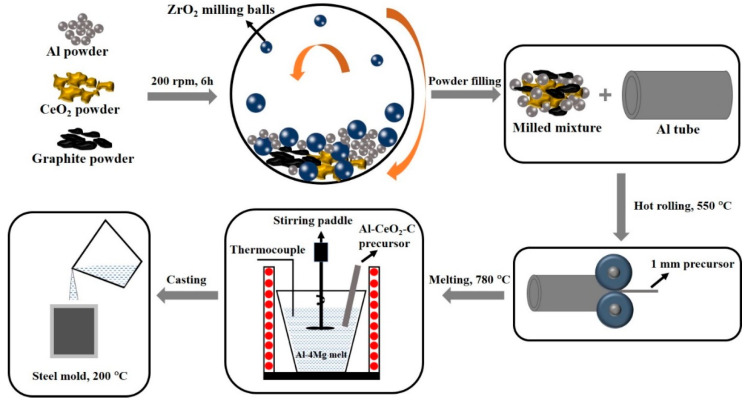
The schematic diagram of the preparation process of Al-Ce-C-O-Mg grain refiner.

A novel rolling-assisted process was used to prepare the Al-Ce-C-O-Mg master alloy. First, 20 g of aluminum powder, 10 g of CeO_2_ powder, and 2 g of carbon powder were dried in a forced-air drying oven. The mixed powders were then placed in a planetary ball mill for ball milling. Here, 32 g ball-milled mixed powder was weighed and sealed into a pure aluminum tube (the aluminum tube weighs 100 g) to prevent powder leakage during the subsequent rolling process. The aluminum tube containing the mixed powder was placed in a box furnace and preheated to 550 °C for 30 min. After holding at this temperature, the material underwent multi-directional rolling, with a 5 min hold at 550 °C between each pass. The multi-directional rolling process involves rolling preheated aluminum tubes containing mixed powders under a rolling mill. Each pass reduces the thickness by 1 mm. After the first rolling pass, the sample is rotated 90° for a second rolling pass, resulting in a final sample thickness of 1 mm. Finally, the aluminum tube containing the mixed powder was rolled to a thickness of 1 mm, forming the “Al-CeO_2_-C precursor”, which was then placed in a drying oven for further preheating and storage. Then, 320 g pure aluminum and 30 g pure magnesium ingots were heated in a pit-type furnace to 780 °C to obtain an aluminum melt. It is important to note that due to combustion losses during high-temperature reactions, a margin of error should be factored into the raw material ratios. The 150 g Al-CeO_2_-C precursor was added to the melt, followed by a 2-h holding treatment. During the holding period, stirring was performed every 20 min. After the reaction was complete, the melt was poured into a steel mold. Following solidification, the Al-Ce-C-O-Mg grain refiner was demolded. The chemical composition of the obtained Al-Ce-C-O-Mg master alloy was Al-1.5Mg-1.2Ce-0.6O-0.3C (wt.%). The preparation process is shown in Figure 1.

In this study, the AZ91 alloy was used to verify the performance of the Al-Ce-C-O-Mg grain refiner. The specific refining experiments referred to previous research [11]. After polishing, the grain refiner samples were subjected to etching using Buswell solution. After polishing, the AZ91 alloy was etched using a 4% nitric acid solution. EBSD analysis was performed using an FEI-XL30 field-emission SEM (Waltham, MA, USA) to observe the grain size evolution. The preparation process for the EBSD samples included mechanical polishing and argon ion polishing. The obtained EBSD data were analyzed using AZtec commercial software (AZtecHKL, https://nano.oxinst.com/products/aztec/aztechkl, accessed on 15 September 2025). The compositional analysis of the grain refiner sample was conducted using inductively coupled plasma spectroscopy (ICP) and oxygen, nitrogen, and hydrogen analysis (ONH-P). An X-ray diffractometer (XRD, Empyrean, Malvern Panalytical, Almelo, The Netherlands) was used to analyze the phase evolution. Metallographic observations were performed on a LEICA DMi8 metallographic microscope (OM) (Wetzlar, Germany). Transmission electron microscopy (TEM) analysis was performed on a JEM-2100 (JEOL Ltd., Akishima, Japan). The room-temperature tensile mechanical properties were tested using an Instron electronic universal testing machine (model 5982) (Norwood, MA, USA) at a tensile rate of 2 mm/min. The tensile samples of the cast alloy were prepared in accordance with the GB/T 228-2002 national standard [19], and each sample’s tensile test was repeated at least twice.

## 3. Results and Discussion

### 3.1. Microstructure Evolution of the Al-Ce-C-O-Mg Master Alloy

**Figure 2:**
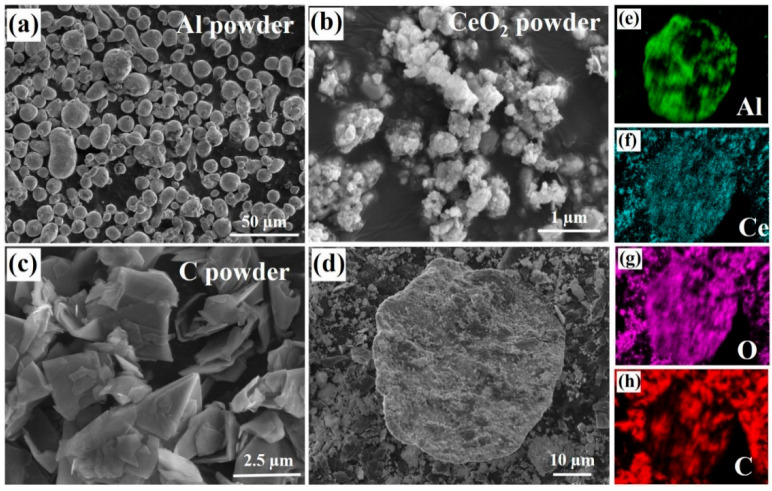
Morphologies of raw powders and ball-milled mixed powders. (**a**) Al powder, (**b**) CeO_2_ powder, (**c**) C powder, (**d**) the mixed powders, (**e**–**h**) the elements distribution of Al, Ce, O, and C in (**d**).

The microstructure of the reaction raw materials are shown in Figure 2. It can be observed that the original Al powder is spherical in shape (particle size 20 μm), while CeO_2_ powder exhibits an irregular polygonal shape (particle size less than 1 μm), and C powder has a flaky morphology, as shown in Figure 2a–c. The morphologies of the mixed powder after 6 h of ball milling are shown in Figure 2d. The spherical aluminum powder undergoes varying degrees of deformation, becoming flaky. This flaky morphology of the Al powder benefits the adsorption of CeO_2_ and C powders, promoting the full integration of the particle phase with the Al powder. As shown in Figure 2d, CeO_2_ and C powders are distributed on the aluminum powder. The EDS surface scanning images of Al, Ce, O, and C elements in Figure 2e–h further confirm the complete integration of Al powder with CeO_2_ and C powders.

**Figure 3:**
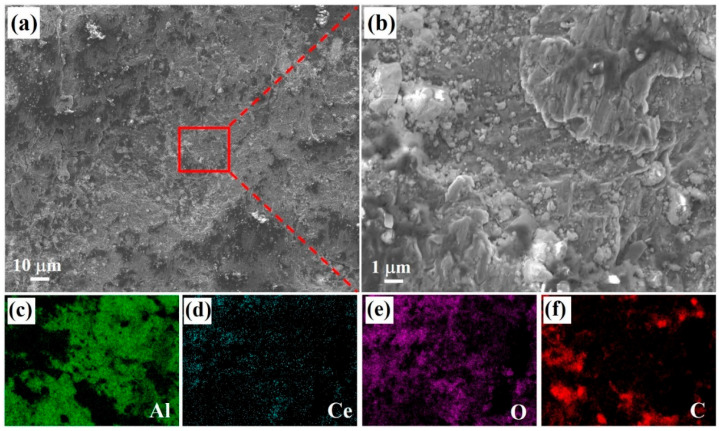
The microstructure of the Al-CeO_2_-C precursor after hot-rolling. (**a**,**b**) Morphologies of the bonding surface of mixed powders and Al tube after hot-rolling; (**c**–**f**) element distribution maps of Al, Ce, O, and C in (**b**).

The pure Al tube filled with mixed powders was rolled indirectly to obtain Al-CeO_2_-C precursor. Figure 3 shows the morphologies of the Al-CeO_2_-C precursor after peeling. The Al powders, CeO_2_ powders, and C powders achieve adequate bonding, as shown in Figure 3a,b. The Al, CeO_2_, and lamellar C powder are welded together at high temperature and pressure. The distribution maps of Al, Ce, O, and C elements reveal that CeO_2_ and C powder are uniformly distributed in the Al block, and no complete Al powder morphologies can be observed. The above results indicate that the hot rolling process can effectively combine Al powder, CeO_2_ powder, and C powder, thereby reducing the gap between the particles. Using an Al-CeO_2_-C precursor fabricated via hot rolling eliminates excessive interparticle voids that are typically present in untreated powder mixtures. This densification of the precursor material effectively mitigates issues encountered during subsequent melting operations, including the tendency of loose powders to float on the melt surface and the poor wettability between the powders and the molten Al matrix.

**Figure 4:**
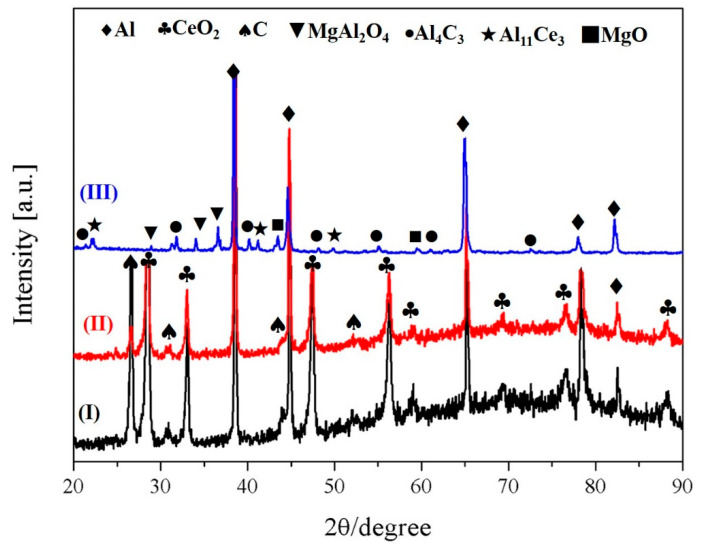
X-ray diffraction patterns: (I) the milled Al, CeO_2,_ and C powders; (II) the Al-CeO_2_-C precursor; (III) the Al-Ce-O-C-Mg master alloy.

Figure 4 presents X-ray diffraction images of the individual milled Al, CeO_2_, and C powders, the Al-CeO_2_–C precursor, and the prepared Al-Ce-C-O-Mg master alloy synthesized via the rolling-assisted process. For the milled powder mixture (sample I) and the hot-rolled precursor (sample II), only the characteristic diffraction peaks corresponding to CeO_2_, carbon (C), and aluminum (Al) were detected. This observation confirms that neither ball milling nor hot rolling steps induced discernible in situ phase transformations. Instead, these processing routes primarily facilitated mechanical bonding between the constituent powders without initiating chemical reactions. In sample III after undergoing the rolling-assisted process at 780 °C, the characteristic peaks of CeO_2_ disappeared and were replaced by diffraction peaks at the angle of 31.28°, 36.83°, 38.52°, 44.83°, 59.37°, and 65.24°, belonging to the (220), (311), (222), (400), (511), and (440) lattice planes of MgAl_2_O_4_, respectively. Meanwhile, the characteristic peaks of Al_11_Ce_3_ and Al_4_C_3_ appeared in the XRD pattern of sample III. The above XRD results showed that reactions generated and produced MgAl_2_O_4_, Al_4_C_3_, and Al_11_Ce_3_ phases after the rolling-assisted process.

**Figure 5:**
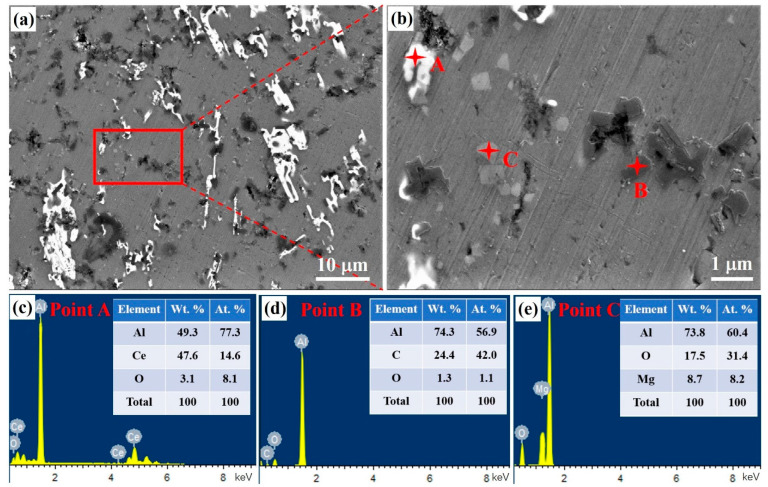
The morphologies of the prepared Al-Ce-O-C-Mg master alloy by the rolling-assistance process. (**a**) SEM image; (**b**) higher-magnification micrograph of the red box in (**a**); (**c**–**e**) EDS point analysis results of points A, B, and C in (**b**).

The SEM microstructure of Al-Ce-O-C-Mg is shown in Figure 5, where the uniform distribution of MgAl_2_O_4_ and Al_4_C_3_ phases is in the Al matrix. Figure 5b shows a higher-magnification micrograph of the area outlined by the red box in Figure 5a. The light gray MgAl_2_O_4_ particles are multilaterally shaped particles with a size of less than 2 μm. The dark gray Al_4_C_3_ particles also assume a polygonal shape, measuring approximately 5 μm in size. Meanwhile, Al_11_Ce_3_ phases (larger than 10 μm) are distributed in the Al matrix. From the EDS point analysis in Figure 5a, it can be concluded that the MgAl_2_O_4_ particles, Al_4_C_3_, and Al_11_Ce_3_ phases exist. These results correspond to the XRD analysis, proving the occurrence of the reaction in the Al-Ce-O-C-Mg system.

### 3.2. Al-Ce-C-O-Mg System Reaction Mechanism

**Figure 6:**
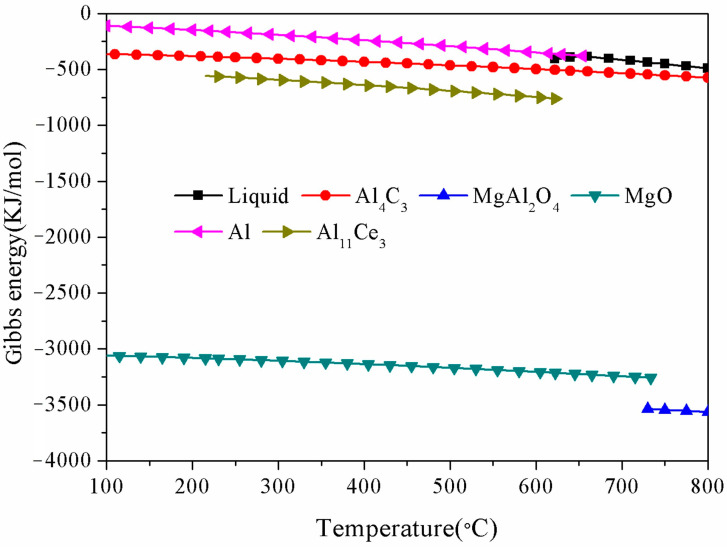
Gibbs free energy of raw material and reaction products of the Al-Ce-O-C-Mg system from 100 °C to 800 °C (the Gibbs free energy parameter was calculated by JMatPro thermodynamic software (13.0)).

**Figure 7:**
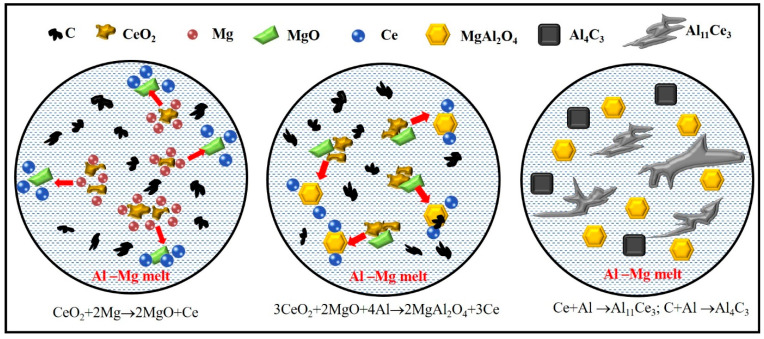
Schematic diagram of the reaction of the Al-Ce-O-C-Mg system in the preparation of the rolling-assisted process.

Jia et al. investigated the phase equilibrium between the reaction products Al_2_O_3_-MgAl_2_O_4_-MgO in the Al-Mg-oxide system by experimentally validating a model that varied the Mg content in the matrix [20]. In general, the necessary condition for forming MgAl_2_O_4_ particles is that the Mg content of the Al-Mg-oxide system is in the range of 0.002-6 wt.%. The Al_2_O_3_ particles can be formed at low Mg content (<0.19 wt.%), while the MgO particles are stable at high Mg content (>7 wt.%) [21]. In this research, the chemical composition of the grain refiner is Al-1.5Mg-1.2Ce-0.6O-0.3C (wt.%), which satisfies the conditions for forming MgAl_2_O_4_ particles. The XRD analysis in Figure 4 also proved the existence of MgAl_2_O_4_ in the Al-Ce-O-C-Mg grain refiner. Figure 6 shows the Gibbs free energy curves of the target products and raw materials in the Al-Ce-O-C-Mg system at different temperatures. Thermodynamically, in combination with the above analysis, the in situ synthesis of MgAl_2_O_4_ particles by the rolling-assisted process was feasible in the Al-Ce-O-C-Mg system. In the present study, the reaction temperature was 780 °C. MgAl_2_O_4_ had the most negative Gibbs free energy value at the synthesis temperature, which meant that the synthesis of MgAl_2_O_4_ was the easiest. Fan et al.’s research indicates that the synthesis reactions of MgO and MgAl_2_O_4_ particles are exothermic. When the chemical reaction is activated, it can generate a large amount of heat in the reaction melt, promoting further reactions between the raw materials. Due to the high-temperature environment, the wettability between Al and C can be enhanced, thereby generating Al_4_C_3_ particles. The Gibbs free energy change of Al_4_C_3_ in Figure 6 also proves its thermodynamic feasibility. Along with the whole reaction process, a large amount of the released active rare-earth [Ce] atoms combined with Al to form phase Al_11_Ce_3_, which XRD detected (Figure 4). Figure 7 shows the synthesis of particles in the Al-Ce-O-C-Mg system. It can be seen that MgAl_2_O_4_ is formed by the reaction of CeO_2_, MgO, and Al, while the reaction of free Ce and C elements with the Al matrix generates Al_11_Ce_3_ and Al_4_C_3_.

### 3.3. Grain Refinement by Al-Ce-O-C-Mg Grain Refiner on AZ91 Alloy

**Figure 8:**
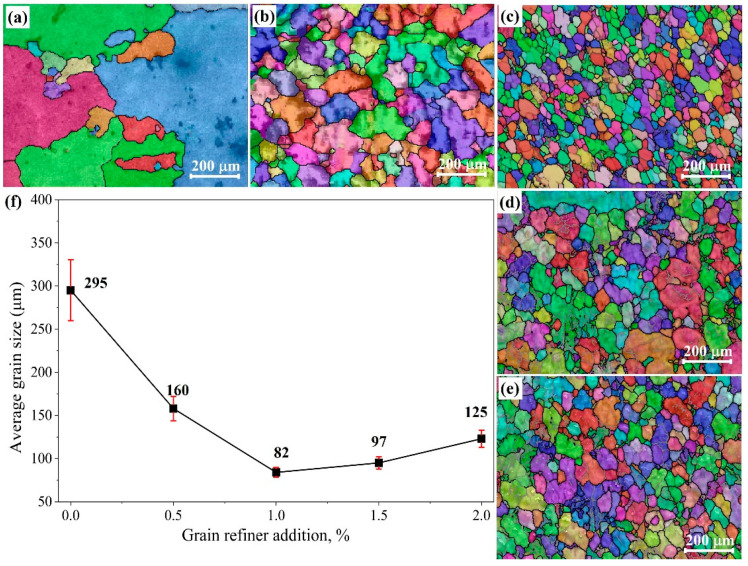
EBSD microstructures of the AZ91 alloys with different amounts of Al-Ce-O-C-Mg grain refiner: (**a**) original AZ91 alloy, (**b**) AZ91 alloy with 0.5 wt.% grain refiner, (**c**) AZ91 alloy with 1.0 wt.% grain refiner, (**d**) AZ91 alloy with 1.5 wt.% grain refiner, and (**e**) AZ91 alloy with 2.0 wt.% grain refiner, (**f**) average grain sizes of the AZ91 alloys with different amounts of Al-Ce-O-C-Mg grain refiner.

**Figure 9:**
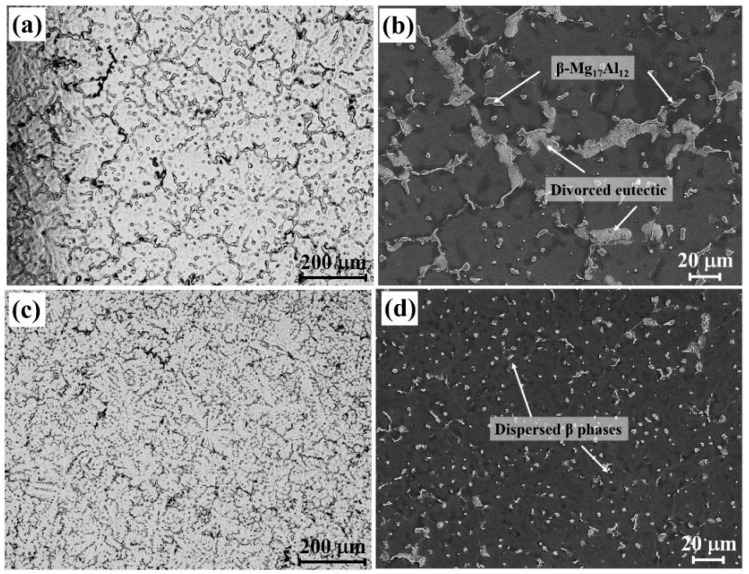
OM and SEM images of as-cast AZ91 alloys: (**a**,**b**) AZ91 alloy without grain refiner; (**c**,**d**) AZ91 alloy with 1.0 wt.% Al-Ce-O-C-Mg grain refiner.

Figure 8 illustrates the EBSD maps of the AZ91 alloy with varying additions of the Al-Ce-O-C-Mg. A pronounced refinement was observed upon the incorporation of the refiner. The grain size of the unmodified AZ91 alloy was approximately 295 μm. The introduction of 0.5 wt.% refiner reduced the grain size to 160 μm, while a further increase to 1.0 wt.% resulted in the finest microstructure, with a grain size of about 82 μm. Partial grain coarsening occurred when the content was increased to 2.0 wt.%, yielding a grain size of 125 μm. This reduction in refinement efficiency at higher additions is attributed to the excessive presence of heterogeneous nucleation sites, which promote agglomeration into larger particle clusters. Such clusters tend to settle during solidification, thereby diminishing their effectiveness as nucleants [22]. Figure 9 presents the OM and SEM images of AZ91 alloys with and without 1.0 wt. % Al-Ce-O-C-Mg. In the refined alloy, the coarse β-Mg_17_Al_12_ phase and the divorced eutectic structure were significantly reduced in size, and their spatial distribution became notably more uniform relative to the unrefined counterpart. These observations indicate that adding the Al-Ce-O-C-Mg grain refiner decreases the matrix grain size and optimizes the morphology and dispersion of the secondary phases. Such microstructural modifications are expected to contribute positively to the overall mechanical performance of the AZ91 alloy [23].

**Figure 10:**
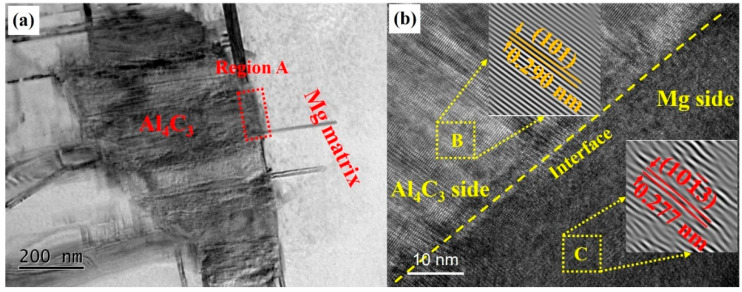
(**a**) TEM microstructure of the sample containing Al_4_C_3_ particles and Mg matrix; (**b**) HRTEM image of region A in (**a**) and IFFT micrographs of region B (for Al_4_C_3_) and region C (for α-Mg).

Previous investigations have identified a clear crystallographic mismatch relationship between MgAl_2_O_4_ and the α-Mg, supporting the role of MgAl_2_O_4_ particles as potent nucleation sites for α-Mg [18,22]. Al_4_C_3_ particles have also been recognized as significant contributors to the refinement of Al-containing Mg alloy. Figure 10a presents TEM images of Al_4_C_3_ particles embedded within the Mg matrix, revealing a distinct and clean interface between the particle and the surrounding α-Mg. An HR-TEM image of region A is shown in Figure 10b, demonstrating the absence of misfit dislocations or interfacial defects, implying strong atomic-level bonding between Al_4_C_3_ and α-Mg. Furthermore, inverse fast Fourier transform (IFFT) analyses carried out on region B (corresponding to Al_4_C_3_) and region C (corresponding to α-Mg) in Figure 10b confirm that there was a specific orientation relationship between the Al_4_C_3_ and α-Mg ((101)_Al4C3_//(1$0\overset{-}{1}3$)_α-Mg_), which further substantiates the nucleation-facilitating role of Al_4_C_3_. In our previous work, we confirmed a specific matching relationship between MgAl_2_O_4_ and Mg. MgAl_2_O_4_ particles can also act as a nucleation site for Mg [24]. Therefore, the Al-Ce-O-C-Mg grain refiner developed in this research contains two types of refining particles, which can effectively achieve grain refinement in AZ91 alloys.

### 3.4. Effect of Al-Ce-O-C-Mg Grain Refiner on the Mechanical Properties of AZ91 Alloy

**Figure 11:**
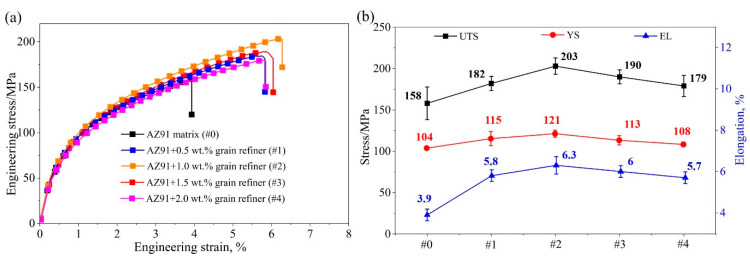
(**a**) Tensile curves and (**b**) mechanical properties of as-cast AZ91 alloys with various Al-Ce-O-C-Mg grain refiners (the black line represents the ultimate tensile strength, the red line represents the yield strength, and the blue line represents the elongation).

Stress–strain curves and tensile mechanical property data of unrefined and refined AZ91 alloys are presented in Figure 11. Here, 0# to 4# represent the original AZ91 alloy and AZ91 alloy with 0.5 wt. %, 1 wt. %, 1.5 wt. %, and 2 wt. % Al-Ce-O-C-Mg, respectively. Adding grain refiner can enhance the tensile mechanical properties of AZ91 alloy. The UTS, YS, and EL of the original AZ91 alloy were 158 MPa, 106 MPa, and 3.9%, respectively. After inoculation, the optimal UTS, YS, and EL of the AZ91 alloy were 203 MPa, 121 MPa, and 6.3%, respectively. When the amount of grain refiner exceeded 1.5 wt.%, the tensile properties tended to decrease, corresponding to the changing trend in grain size, as shown in Figure 8.

**Figure 12:**
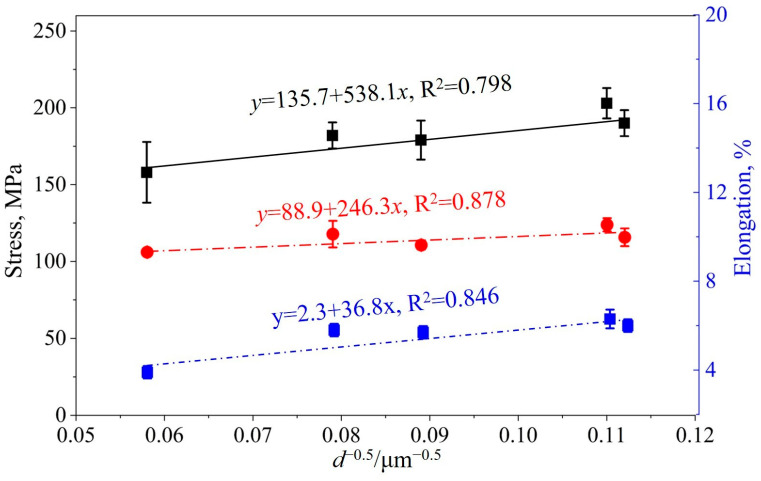
Relationship between UTS, YS, EL, and the inverse square root of grain size for AZ91 alloy (the black solid line represents the ultimate tensile strength, the red dashed line represents the yield strength, and the blue dashed line represents the elongation).

Microstructural refinement can enhance the alloy’s strength and elongation, which can be correlated. The relationship between the yield strength and grain size can be described by the Hall–Petch equation [25]:

σ_s_ = σ_0_ + *kd*^−1/2^ (1)

where σ_s_ is the yield strength, σ_0_ is the lattice friction force, *k* is the Hall–Petch coefficient, and *d* is the grain size of the AZ91 alloy. As illustrated in Figure 12, the UTS, YS, and EL were plotted against the negative reciprocal square root of the average grain size. Linear regression of the experimental data revealed that all three mechanical parameters exhibited near-linear dependence, per the Hall–Petch relation, with coefficients of determination (*R*^2^) of 0.798, 0.878, and 0.846, respectively. These results unequivocally indicate that the tensile deformation behavior of the AZ91 alloy is governed by grain size. The analysis confirms that grain boundary strengthening, arising from microstructural refinement, plays a predominant role in enhancing both the strength and ductility of the alloy.

## 4. Conclusions

This work synthesized an Al-Ce-O-C-Mg grain refiner via a novel rolling-assisted process and applied it to as-cast AZ91 Mg alloy. The effects on grain size, tensile properties, and the refinement and strengthening mechanisms were systematically investigated. The key findings are as follows:

(1) The Al-Ce-C-O-Mg grain refiner prepared by the rolling-assisted process contains two types of effective refining particles—MgAl_2_O_4_ and Al_4_C_3_. These particles are the key to grain refinement for AZ91 alloy.
(2) Incorporating the Al-Ce-C-O-Mg grain refiner into the AZ91 alloy matrix markedly enhanced grain refinement efficacy. An additional level of 1.5 wt.% was the most effective, producing an approximately 72% reduction in the average grain size relative to the unmodified alloy. This microstructural refinement was accompanied by substantial improvements in tensile performance: the UTS increased from 158 MPa to 203 MPa, YS rose from 104 MPa to 121 MPa, and EL improved from 3.9% to 6.3%.
(3) The MgAl_2_O_4_ and Al_4_C_3_ particles present in the grain refiner possess favorable lattice matching with the α-Mg matrix. In particular, Al_4_C_3_ displays a crystallographic orientation relationship with Mg, indicating a well-bonded interface. MgAl_2_O_4_ and Al_4_C_3_ particles can act as potent heterogeneous nucleation sites for α-Mg during solidification, facilitating efficient nucleation and contributing to substantial grain refinement.

## Data Availability

The original contributions presented in this study are included in the article. Further inquiries can be directed to the corresponding author.
